# Reversible Photochromic Reactions of Bacteriorhodopsin from *Halobacterium salinarum* at Femto- and Picosecond Times

**DOI:** 10.3390/molecules29204847

**Published:** 2024-10-13

**Authors:** Olga Smitienko, Tatyana Feldman, Ivan Shelaev, Fedor Gostev, Arseniy Aybush, Dmitry Cherepanov, Victor Nadtochenko, Mikhail Ostrovsky

**Affiliations:** 1 Emanuel Institute of Biochemical Physics of the Russian Academy of Sciences, Kosygin St., 4, Moscow 119334, Russia; feldmantb@mail.ru (T.F.); ostrovsky3535@mail.ru (M.O.); 2 Department of Biology, Lomonosov Moscow State University, Leninskie Gory, 1, Moscow 119991, Russia; tscherepanov@gmail.com; 3 Moscow Center for Advanced Studies, Kulakova Str. 20, Moscow 123592, Russia; shelaevivan@gmail.com (I.S.); boatsween@yandex.ru (F.G.);; 4 N.N. Semenov Federal Research Center for Chemical Physics of the Russian Academy of Sciences, Kosygin St., 4, Moscow 119991, Russia; 5 Department of Chemistry, Lomonosov Moscow State University, Leninskie Gory, 1, Moscow 119991, Russia

**Keywords:** rhodopsins, bacteriorhodopsin, retinal, photochemistry, femtosecond spectroscopy

## Abstract

The operation of bacteriorhodopsin (*BR*) from the archaeon *Halobacterium salinarum* is based on the photochromic reaction of isomerization of the chromophore group (the retinal protonated Schiff base, RPSB) from the all-*trans* to the 13-*cis* form. The ultrafast dynamics of the reverse 13-*cis* → all-*trans* photoreaction was studied using femtosecond transient absorption spectroscopy in comparison with the forward photoreaction. The forward photoreaction was initiated by photoexcitation of *BR* by pulse I (540 nm). The reverse photoreaction was initiated by photoexcitation of the product *K*_590_ at an early stage of its formation (5 ps) by pulse II (660 nm). The conversion of the excited *K*_590_ to the ground state proceeds at times of 0.19, 1.1, and 16 ps with the relative contributions of ~20/60/20, respectively. All these decay channels lead to the formation of the initial state of *BR* as a product with a quantum yield of ~1. This state is preceded by vibrationally excited intermediates, the relaxation of which occurs in the 16 ps time range. Likely, the heterogeneity of the excited state of *K*_590_ is determined by the heterogeneity of its chromophore center. The forward photoreaction includes two components—0.52 and 3.5 ps, with the relative contributions of 91/9, respectively. The reverse photoreaction initiated from *K*_590_ proceeds more efficiently in the conical intersection (CI) region but on the whole at a lower rate compared to the forward photoreaction, due to significant heterogeneity of the potential energy surface.

## 1. Introduction

**Figure 1:**
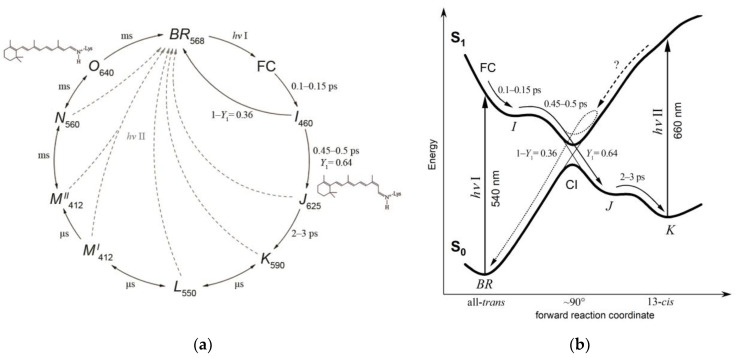
Photoactivated processes of *BR*. (**a**) Photocycle of *BR* [5,6,7,8,9], the reverse photoreactions are shown by dotted curves; subscripts denote the wavelength of absorption maxima. (**b**) Structure of potential energy surfaces (PESs) of S_0_ and S_1_ electron levels of *BR* participating in the forward photoreaction. The reaction coordinate is represented by reaction vibrational modes, and all-*trans*, 13-*cis,* and intermediate forms of RPSB are marked. FC is the Franck–Condon state, CI is the conical intersection.

Bacteriorhodopsin (*BR*) is a transmembrane protein of the halophilic archaeon *Halobacterium salinarum*, the first to be discovered [1] and the best-studied type 1 (microbial) rhodopsin. *BR* performs a photoenergetic function through an active light-dependent proton transport across the cell membrane and is often used as a model system to study the molecular mechanisms of light energy conversion in biological systems. *BR* function is based on the photochemical reaction of isomerization of the chromophore group, the retinal protonated Schiff base (RPSB), from the all-*trans* to the 13-*cis* form [2,3,4]. The photoreaction is accompanied by a series of transformations of the *BR* molecule closed in a photocycle. At different stages of the photocycle in the time range from femto- to milliseconds, the excited state *I*_460_ and intermediate products *J*_625_, *K*_590_, *L*_550_, *M*^I^_412_, *M*^II^_412_, *N*_560_, and *O*_640_ are formed (Figure 1a) [5,6,7]. The result of the photocycle is proton transport from the cytoplasm to the external environment, including the main stages: (i) deprotonation of RPSB and protonation of the primary acceptor Asp85 at the stage of *M*^I^_412_ formation; (ii) release of a proton into the external environment at the stage of *M*^II^_412_ formation; (iii) protonation of RPSB at the stage of *N*_560_ formation; and (iv) proton capture from the cytoplasm at the stage of *O*_640_ formation. During the *N*_560_ → *O*_640_ transition, RPSB undergoes thermal isomerization into the initial all-*trans* form.

The photocycle is initiated only from the all-*trans* form of RPSB, the content of which reaches 90–95% in the light-adapted state of *BR* (*BR*_568_), while 5–10% of the molecules contain RPSB in the 13-*cis* form [10,11], the proportion of which in the dark-adapted state of *BR* reaches 50–60% due to thermal isomerization of RPSB. 

The *BR* photoreaction occurs in the femto- and early picosecond time range, starting from the Franck–Condon state (FC) and ending with the formation of the *K*_590_ product, the lifetime of which is in the microsecond range [12,13,14,15,16,17,18,19,20,21]; further processes of the photocycle are dark (Figure 1a). The photoisomerization of RPSB occurs in the excited *I*_460_ state in the coherent regime and can be described as the evolution of a set of coherent excited vibrational states (wave packet) first along the S_1_ and then along the S_0_ potential energy surfaces (PESs) in accordance with the reaction coordinate (Figure 1b). The S_1_ → S_0_ conversion occurs through the multidimensional conical intersection (CI) region of S_1_/S_0_ PESs [22] with a quantum yield of *J*_625_ formation as high as 0.64 [23]. 

The initial dynamics of the wave packet are determined by the relaxation of RPSB along the totally symmetric (C–C and C=C) vibrational modes, resulting in the formation of *I*_460_ from the FC state in 0.1–0.15 ps [24,25,26]. Thus, *I*_460_ still contains RPSB in the all-*trans* configuration but with inverted C-C and C=C bonds, which has been confirmed both experimentally [27] and theoretically [24]. The reaction dynamics of S_1_ → S_0_ conversion are strongly influenced by the presence of a small barrier to the decay of the excited state of S_1_ due to the interaction with the S_2_ PES, which is reflected by the three-state model (S_0_, S_1_, and S_2_) [3,14,21,24,28,29] (Figure 1b). Then the wave packet begins to move along the reaction vibrational modes—hydrogen out-of-plane (HOOP), and torsional vibrations [17,21,26,30]. During this movement, the wave packet overcomes the barrier at the S_1_ PES in 0.45–0.5 ps, reaches the CI region, and after dividing into two subpackets, passes to the S_0_ PES of the primary reaction product *J*_625_, containing RPSB in the 13-*cis* form, or to the S_0_ PES of the *BR*_568_ initial state [12,13,16,27,31,32]. The structure of RPSB in the product *J*_625_ was identified by time-resolved resonance Raman spectroscopy [33] and ultrafast spectroscopy [27]. The product *J*_625_ is converted with a characteristic time of 2–3 ps into the next product *K*_590_ with a more relaxed 13-*cis* RPSB [33]. The formation of the product *K*_590_ completes the process of converting the energy of a quantum of light into chemical energy in the form of conformational changes in the protein, which are subsequently used for proton transfer.

*BR*, as well as other type 1 rhodopsins and RPSB in solution, is characterized by an additional non-reactive excited-state decay channel with a picosecond lifetime [34,35]. This is associated with branching of the reaction pathway in the FC state [36,37,38] or with the initial heterogeneity of the protein and/or chromophore [14,15,18,37,39]. The latter assumption was confirmed for a number of type 1 rhodopsins [39,40], including *BR* [18].

Rhodopsins are known to have photochromic properties [8,9,23,38,41,42,43,44,45,46,47,48,49,50,51]. The absorption of the second quantum of light by the intermediate of the photocycle can initiate the reverse 13-*cis* → all-*trans* photoisomerization of RPSB, which in many cases proceeds with the formation of the initial state of *BR*_568_ (Figure 1a) [8]. The ability to photochromism is used in nature in a number of rhodopsins to perform certain physiological functions. For example, sensory rhodopsin of the cyanobacterium *Anabaena sp.* (ASR) regulates the transcription of genes of the photosynthetic apparatus [52], sensory rhodopsin I of *H. salinarum* (SRI) and channel rhodopsin of the unicellular green alga *Chlamydomonas reinhardtii* (ChR2) carry out negative phototaxis [53,54,55], and bistable rhodopsins of type 2 (animal rhodopsins) perform visual and non-visual photoreceptor functions [56].

The study of photochromic reactions initiated from different stages of photoconversion is one of the experimental approaches to studying the mechanism of photochemical reaction and subsequent processes of dark relaxation of rhodopsins. The examination of the reverse photochromic reaction from the primary intermediate state can provide new knowledge about ultrafast photoisomerization of retinal chromophores in different protein environments. The reverse phototransition *K*_590_ → *BR*_568_ initiated at the early stages of the photocycle, when large-scale changes in the protein part of the molecule have not yet occurred, is of interest. This phototransition was studied using low-temperature spectrophotometry [8,46,57,58,59,60,61], time-resolved photoelectrometry [62], and time-resolved spectroscopy upon excitation of *K*_590_ in the pico- [44,45,50,63] and nanosecond [15,23,47] time range. The *K*_590_ → *BR*_568_ phototransition was shown to occur with a high quantum yield of 0.8–1 [8,23,44,47,60] without the formation of detectable intermediate products. However, it remains unclear whether the protein state observed upon relaxation of the system in the time interval of 0.1–10 ns is identical to the “hot” *K*_590_ state that appears immediately after excitation. The differences may be in both the conformation of the RPSB and the protein. Measuring the quantum yield of the reverse photoreaction initiated immediately after the all-*trans* → 13-*cis* transition is an important complement to the measurements already performed.

In this work, we investigated the dynamics of the *K*_590_ → *BR*_568_ reverse phototransition at the early stage of *K*_590_ formation 5 ps after the initiation of the forward photoreaction. The work was carried out using the pump-probe and pump-pump-probe methods of femtosecond transient absorption spectroscopy with a time resolution of 25 fs and probing in the spectral range of 400–740 nm. It was shown that the dynamics of the reverse photoreaction of *BR* is nonexponential with the slow component of S_1_ → S_0_ conversion completing only at ~16 ps after excitation. This most likely indicates a strong heterogeneity of the chromophore center of the *K*_590_ product, in contrast to the chromophore center of the initial state of *BR*_568_. The kinetics of reverse *BR* photoreaction observed in this work are similar to those measured at a delay time of 60 ps in [45]. A new method of data analysis allowed us to identify an unknown intermediate of the reverse photoreaction converting to *BR*_568_ at ~16 ps. This method can be applied to study the dynamics of reverse photoreactions of other rhodopsins at the earliest stages of their photoconversion.

## 2. Results

### 2.1. Forward Photoreaction

**Figure 2:**
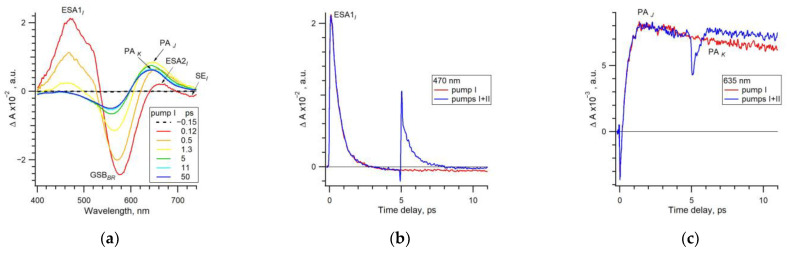
Forward photoreaction of *BR*. (**a**) Transient absorption spectra Δ*A*^I^(λ, *t*) recorded at characteristic time delays upon pumping by pulse I; (**b**,**c**) Kinetic curves of photoinduced *BR* absorption Δ*A*^I^(λ, *t*) and Δ*A*^I+II^(λ, *t*) recorded upon pumping by pulse I and pulses I + II, respectively, at probing wavelengths of 470 (**b**) and 635 (**c**) nm.

The forward photoreaction was initiated by pumping *BR*_568_ with a pulse I peaking at 540 nm. The *BR* photoinduced absorption Δ*A*^I^(λ, *t*) results were recorded at time delays of up to 11 ps and at a time delay of 50 ps. Figure 2a shows the difference spectra Δ*A*^I^(λ, *t*) that characterize the forward photoreaction of *BR* and have already become classical [13,35,46,64]. At a time delay of 0.12 ps, short-wavelength (ESA1*_I_*) and long-wavelength (ESA2*_I_*) absorption and stimulated emission (SE*_I_*) signals from the excited state *I*_460_ appear in the spectral regions of 400–550 nm (ESA1*_I_*) and >640 nm (ESA2*_I_* and SE*_I_*) (Figure 2a, red curve). Over the next 1–1.5 ps, these signals are replaced by an absorption band in the 600–740 nm region (PA*_J_*) (Figure 2a, yellow curve), which reflects the S_1_ → S_0_ transition from *I*_460_ to the primary product *J*_625_, which contains isomerized RPSB in the 13-*cis* form. In the picosecond time range, the absorption band of *J*_625_ in the difference spectra shifts to the short-wavelength region and becomes less intense, which characterizes the formation of the next product *K*_590_ (PA*_K_*) as a result of vibrational and conformational relaxation (Figure 2a, light blue and blue curves).

In the range of 500–650 nm, a negative signal is observed, associated with the ground state bleaching of *BR*_568_ (GSB*_BR_*) (Figure 2a). This band coincides in shape with the inverted absorption spectrum of *BR*_568_. At a time delay of 0.12 ps, the intensity of the GSB*_BR_* band in the difference spectrum is a maximum, and at a 5 ps delay, when the photoreaction is complete, the intensity of this band drops significantly. This is due to factors: (i) the formation of the products *J*_625_ and *K*_590_, absorption of which occurs in the same spectral region as the GSB*_BR_* band, and (ii) the return of some of the excited molecules to the initial state of *BR*_568_ during the photoreaction. The kinetic curves Δ*A*^I^(470 nm, *t*) (Figure 2b, red curve) and Δ*A*^I^(635 nm, *t*) (Figure 2c, red curve) reflect the processes described above.

**Scheme 1:**
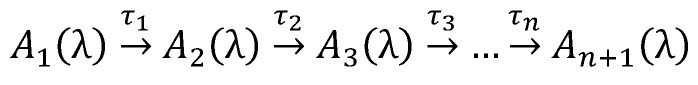
Sequential kinetic model of the forward and reverse photoreactions of *BR*.

**Figure 3:**
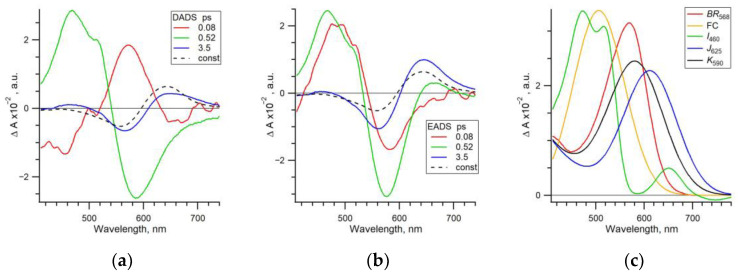
Forward photoreaction of *BR*. (**a**) Exponential decay-associated difference spectra (DADS); (**b**) Evolution-associated difference spectra (EADS); difference spectrum Δ*A*^I^(λ, 50 ps) was used as a final spectrum (const); (**c**) Absorption spectra of the states participating in the forward *BR* photoreaction: *BR*_568_, FC, *I*_460_, *J*_625_, and *K*_590_; the spectra were normalized in accordance with the extinction coefficients.

Decomposition of the spectral-temporal matrix Δ*A*^I^(λ, *t*) at delays up to 50 ps into a linear combination of discrete exponential functions in accordance with Equation (1) (see Section 4) made it possible to determine the times τ_0_–τ_2_ characterizing the forward photoreaction (Table 1). Exponential decay-associated difference spectra (DADS) and the relevant evolution-associated difference spectra (EADS) of the successively evolving intermediates of the sequential kinetic model (Scheme 1) are presented in Figure 3.

Calculated lifetimes τ_0_ = 0.08 ps, τ_1_ = 0.52 ps, and τ_2_ = 3.5 ps (Table 1) are in good agreement with the data obtained previously [12,14,16,18,19,20,31,65]. The time τ_0_ reflects the dynamics of the Stokes shift in the course of the formation of *I*_460_ from the FC state. As can be seen from Figure 3a (red curve), with this characteristic time, the ESA1*_I_* and ESA2*_I_* bands shift to the short-wavelength region, and the SE*_I_* band shifts to the long-wavelength region, which reflects the motion of the wave packet along the S_1_ PES and the accompanying process of vibrational relaxation. The time τ_1_ characterizes the *I*_460_ decay along the reactive pathway (Figure 1b). The DADS of the respective absorption changes (Figure 3a, green curve) demonstrates the almost complete disappearance of the ESA1*_I_* band in the region of 400–550 nm and an increase in PA*_J_* (600–700 nm) and *BR*_568_ absorption (560–600 nm). The slow characteristic time τ_2_ reflects a minor residual disappearance of the ESA1*_I_* band, a drop in the PA*_J_*, and an increase in the PA*_K_* and *BR*_568_ absorption (Figure 3a, blue curve), which indicates both the non-reactive path of the *I*_460_ → *BR*_568_ transition [18,31] and the *J*_625_ → *K*_590_ transition. Most likely, a vibrationally excited *BR*′ state is formed during the photoreaction, and the relaxation of this state takes several picoseconds, as in the case of the *J*_625_ → *K*_590_ transition.

Figure 3b shows the respective EADS calculated by the sequential kinetic Scheme 1, which reflect signals from the FC state, *I*_460_, *J*_625_, and *K*_590_, on which the GSB*_BR_* band is superimposed. The decomposition of EADS into Gaussians and the GSB*_BR_* band (see Supplementary Materials, Figure S1) made it possible to obtain the absorption spectra of these states (Figure 3c). This also allowed us to estimate the contributions of the reactive and non-reactive pathways to the overall dynamics of the excited state decay based on the drop in the ESA1 signal (see Supplementary Materials, Figure S1a,b). The contributions of the reactive and non-reactive pathways were 91% and 9%, respectively (Table 1). The slow component of the excited state decay in *BR* was observed earlier both from transient absorption [14,18,19,20,27,65] and fluorescence [16] signals.

### 2.2. Reverse Photoreaction

**Figure 4:**
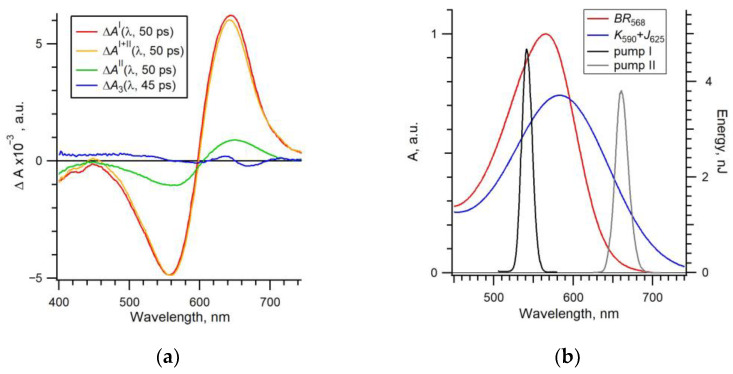
(**a**) Difference absorption spectra of *BR* recorded after pumping by pulses I (Δ*A*^I^(λ, 50 ps)), I + II (Δ*A*^I+II^(λ, 50 ps)), and II (Δ*A*^II^(λ, 50 ps)) at a time delay of 50 ps; difference spectrum Δ*A*_3_(λ, 45 ps) calculated based on Equation (S7). In the spectral regions of pulses I (530–590 nm) and II (650–710 nm), the spectra were completed using model curves. (**b**) Absorption spectra of *BR*_568_ (red curve) and *K*_590_ (with contribution of *J*_625_) at a time delay of 5 ps (blue curve) normalized in accordance with the extinction coefficients (left axis); spectra of pump pulses I (black curve) and II (grey curve) (right axis).

The reverse photoreaction was initiated by pulse II centered at 660 nm by pumping the *K*_590_ product at an early stage of its formation, 5 ps after the action of pulse I. It is worth noting that, since the *J*_625_ → *K*_590_ transition is not completed at this time delay, the reverse photoreaction is partially initiated from the *J*_625_ product. The Δ*A*^I+II^(λ, *t*) signals were recorded at time delays of up to 11 ps (Figure 2b,c, blue curves) and at a time delay of 50 ps (Figure 4a, orange curve). Figure 4b shows the absorption spectra of *BR*_568_ (red curve) and the *K*_590_ product with an admixture of the *J*_625_ product at a time delay of 5 ps (blue curve) normalized in accordance with the extinction coefficients. The absorption spectrum of *K*_590_ + *J*_625_ was calculated by the difference spectrum Δ*A*^I^(λ, 5 ps) (Figure 2a, green curve) using the GSB*_BR_* band (see Supplementary Materials, Figure S1b,c). It is characterized by a maximum at 583 nm, a width of 1.4 times greater than the width of the *BR*_568_ absorption spectrum, and an extinction that is only 0.74 of the *BR*_568_ extinction.

Pulse II was centered at 660 nm for optimal excitation of *K*_590_ and minimal excitation of *BR*_568_. However, pulse II also excites a small fraction of *BR*_568_ molecules, initiating the forward photoreaction. To account for this process, the Δ*A*^II^(λ, 50 ps) spectrum was recorded under the action of only pulse II (Figure 4a, green curve).

Comparison of the kinetic curves Δ*A*^I^(λ, *t*) and Δ*A*^I+II^(λ, *t*), presented in Figure 2b,c, showed that at the time delays before the action of pulse II, these curves practically repeat each other. At later times, changes in the difference signal are observed. Comparison of the signals Δ*A*^I^(λ, 50 ps), Δ*A*^I+II^(λ, 50 ps), and Δ*A*^II^(λ, 50 ps) (Figure 4a) allowed us to track the changes in the *BR* photoinduced absorption at the time delays, when all primary processes have already been completed and the difference spectra consist only of the positive PA*_K_* and the negative GSB*_BR_* bands. The action of pulse II leads to a slight drop in the PA*_K_* band (Figure 4a, orange curve).

**Figure 5:**
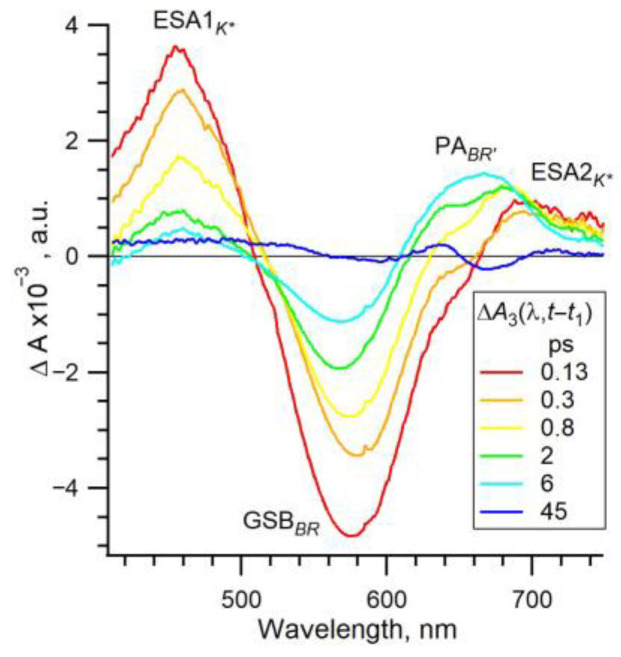
Reverse photoreaction of *BR*. Difference spectra Δ*A*_3_(λ, *t*—*t*_1_) reflecting the formation of the excited state *K*_590_* and the products other than *BR*_568_ and calculated on the basis of Equation (S6) at time delays *t*—*t*_1_ of 0.13–6 ps and on the basis of Equation (S7) at a time delay of 45 ps relative to pulse II. In the spectral regions of pulses I (530–590 nm) and II (650–710 nm), the spectra were completed using model curves.

The absorption difference spectra Δ*A*^I+II^(λ, *t*) after the action of pulse II reflect the signals from three *BR* ensembles: (1) *BR*_568_ molecules excited by pulse I but not affected by pulse II; (2) *BR*_568_ molecules excited only by pulse II; (3) *K*_590_ molecules (and partially *J*_625_) excited by pulse II. The time evolution of ensembles (1) and (2) is determined by the dynamics of the forward photoreaction with time delays *t* and *t*—*t*_1_ relative to pulse I, respectively, where *t*_1_ = 5 ps, and the evolution of ensemble (3) is determined by the dynamics of the reverse photoreaction *K*_590_ → *BR*_568_ with a time delay *t*—*t*_1_ relative to pulse II. The absorption changes of the three *BR* ensembles are described by Equations (S3), (S5), and (S6) (see Supplementary Materials). The absorption dynamics of the third ensemble, Δ*A*_3_(λ, *t*—*t*_1_), characterizes the reverse photoreaction (Figure 5).

**Figure 6:**
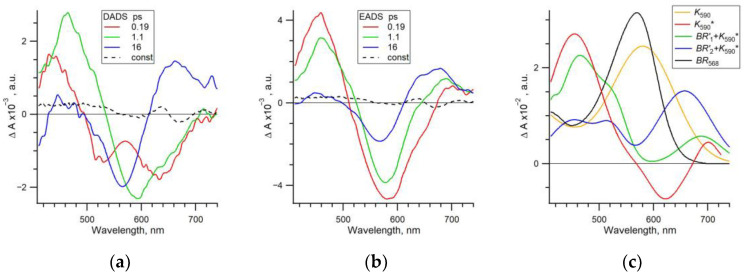
(**a**) Exponential decay-associated difference spectra (DADS); (**b**) The evolution-associated difference spectra (EADS); difference spectrum Δ*A*_3_(λ, 45 ps) calculated on the basis of Equation (S7) was used as a final spectrum (const); (**c**) Absorption spectra of the states participating in the reverse *BR* photoreaction: *K*_590_, *K*_590_*, *BR*′_1_ (with significant contribution from *K*_590_*), *BR*′_2_ (with small contribution of *K*_590_*), and *BR*_568_.

Decomposition of the spectral-temporal matrix Δ*A*_3_(λ, *t*—*t*_1_) into a linear combination of discrete exponential functions in accordance with Equation (1) (see Section 4) made it possible to determine the times characterizing the reverse photoreaction of *BR* (Table 1). The exponential decay-associated difference spectra (DADS) (Figure 6a) and the difference spectra *A*_k_(λ) of intermediate products (EADS) (Figure 6b) of the sequential kinetic model (Scheme 1) were also calculated.

In the dynamics of the reverse *BR* photoreaction, three transitions with characteristic times τ′_1_ = 0.19 ps, τ′_2_ = 1.1 ps, and τ′_3_ = 16 ps were revealed (Table 1). The corresponding DADS and EADS together with the final spectrum Δ*A*_3_(λ, 45 ps) are shown in Figure 6a and b, respectively. The EADS of the fastest intermediate resolved for delays shorter than τ′_1_ (Figure 6b, red curve) reflects the spectrum of relaxed excited state of *K*_590_ (*K*_590_*)—an analogue of the excited state *I*_460_—relative to the GSB*_BR_* band. This spectrum, similar to the spectrum of *I*_460,_ contains an intense ESA1_K_* band and a small ESA2_K*_ band, which are typical for the excited state of RPSB in rhodopsins. The FC state could not be resolved in these measurements. The EADS of two following intermediates evolving with the characteristic times τ′_2_ and τ′_3_ also contain the absorption band of *K*_590_*, which indicates the heterogeneity of the excited state. The complete disappearance of the absorption band in the 460 nm region, characterizing the excited state S_1_, is observed at the characteristic time τ′_3_ synchronously with the disappearance of the absorption band in the 620–700 nm region, related to the vibrationally excited state of the intermediate product *BR*′. The relative distribution of the intramolecular S_1_ → S_0_ conversion through the timescales τ′_1_, τ′_2_, and τ′_3_ was estimated from the ESA1_K_* signal by decomposing the EADS into Gaussian components (see Supplementary Materials, Figure S2) and was found to be 20/60/20, respectively (Table 1). 

The decomposition of EADS into Gaussians also allowed us to characterize the spectra of the different states involved in the reverse photoreaction: *K*_590_* and the intermediate products *BR*′_1_ and *BR*′_2_ (Figure 6c). The absorption spectrum of *K*_590_* (Figure 6c, red curve) acquired from EADS (0.19 ps) includes a significant contribution from the SE signal, which manifests itself as a broad negative band in the 600–660 nm region. At longer delays, an unknown quantity of the ground state *BR*_568_ was produced via the S_1_ → S_0_ conversion in the course of the reverse photoreaction. For this reason, the spectra of the two subsequent kinetic intermediates, *BR*′_1_ and *BR*′_2_, were obtained by decomposing the respective EADS into Gaussian components (see Supplementary Materials, Figure S2b,c). These spectra include a contribution of the *K*_590_* spectrum (Figure 6c, green and blue curves). The absorption changes Δ*A*_3_(620–700 nm, *t*) (Figure 5) reflect both the stimulated emission of the *K*_590_* excited state and the absorption of the vibrationally excited product *BR*′. Quantitative analysis in the 660 nm region is also hampered by the light scattering of the pulse II. For this reason, the available data do not allow a reliable determination to what extent the kinetic intermediates *BR*′_1_ and *BR*′_2_ correspond to different electronic states of the product *BR*′. It can be assumed that the intermediate state *BR*′ is an ensemble of several vibrationally excited precursors of *BR*_568_, analogs of the *J*_625_ state in the forward photoreaction. The character of the DADS curves in the long-wavelength spectral region indicates that the final vibrational relaxation *BR*′ → *BR*_568_ occurs with a characteristic time τ′_3_. 

The spectrum Δ*A*_3_(λ, 45 ps) at the final delay (Figure 5, blue curve; Figure 6a,b, dashed lines) with experimental accuracy is a zero line, which indicates the photoconversion *K*_590_* → *BR*_568_ with a quantum yield close to 1. The obtained value of the quantum yield refines the one (0.8) we calculated earlier in work [44]. Thus, for the reverse photoreaction initiated from the *K*_590_ product, as well as for the forward photoreaction, the presence of several pathways of the excited state decay in the femto- and early picosecond time ranges is characteristic, and all these pathways are reactive.

## 3. Discussion

In this work, the transient absorption dynamics of the *BR* reverse photoreaction initiated from the *K*_590_ product at an early stage of its formation was investigated. The decomposition of the spectral-temporal matrices in the 400–740 nm region into discrete exponential functions (see Section 4, Equation (1)) made it possible to identify model-independent DADS of the three transitions with characteristic times of 0.19, 1.1, and 16 ps (Figure 6a, Table 1). Assuming that the absorption dynamics can be approximated by a sequential kinetic model (Scheme 1), we calculated the EADS of three kinetic intermediates (Figure 6b). All intermediates demonstrated a positive absorption at 460 nm, which is a characteristic feature of the excited state of rhodopsins. Decomposition of EADS into Gaussian functions, together with the linear spectrum of GSB*_BR_* (see Supplementary Materials, Figure S2), allowed us to estimate absorption spectra of the intermediates (Figure 6c). 

The spectrum of excited state *K*_590_* resolved at time delays < 0.19 ps (Figure 6c, red curve) differs from the spectrum of *I*_460_ (Figure 3c, green curve) in several aspects: the main band of *K*_590_* is blue-shifted to 460 nm relative to the maximum at 474 nm of the *I*_460_ band in the difference spectra; the satellite band at 520 nm is not pronounced; and there is a large negative SE signal at 620 nm, whereas the SE of the *I*_460_ state is observed in the far-red region > 720 nm. 

The S_1_ → S_0_ conversion of the *K*_590_* excited state is kinetically heterogeneous. The fastest transition at τ_1_′ = 0.19 ps results in a decrease of the *K*_590_* absorption at 460 nm by ~20% (Table 1, Figure S2a) and to an appearance of the primary vibrationally excited product *BR*′ with the band maximum at 690 nm (Figure 6c, green curve). The next transition at τ_2_′ = 1.1 ps is accompanied by a further decrease of the *K*_590_* absorption by ~60% (Figure S2b) and enhancing the product *BR*′ absorption with a maximum at about 660 nm (Figure 6c, blue curve). Still, there remains some residual *K*_590_* absorption with a relative amplitude of ~20% (Figure S2c). The slowest transition at τ_3_′ = 16 ps leads both to the complete decay of *K*_590_* absorption and to the evolution of *BR*′ to the all-*trans BR*_568_ ground state (Figure 6b, dashed curve). 

The mechanism of the S_1_/S_0_ surfaces crossing in photochemical reactions of various rhodopsins is a matter of long discussion [66,67]. One of the controversial issues is whether the motion along the PES in the CI region is diffusive (overdamped) or inertial. In the case of a diffusive mechanism, the quantum yields of 13-*cis* and all-*trans* products do not depend on whether the reaction proceeds in the forward or reverse direction. If we designate the quantum yield of the 13-*cis* state for the *trans* → *cis* transition (the forward *BR* reaction) as *Y*_1_, and the quantum yield of the *trans* state for the 13-*cis* → all-*trans* transition (the reverse *BR* reaction) as *Y*_2_, then in the case of the diffusion mechanism, the relation *Y*_1_ + *Y*_2_ = 1 is fulfilled [68]. In the alternative case of an inertial mechanism, the quantum yields of 13-*cis* and all-*trans* products depend on the direction of the reaction. The reason for this is that when passing through the thin CI region by the inertial mechanism, the torsional momentum of the nuclear motion is preserved, and this determines the direction of the system’s motion along the reaction coordinate [68,69,70,71]. In a simplified one-dimensional model of the CI region, the system can move along any of the two diabatic terms (thin solid lines in Figure 1b) forming either the product *J*_625_ or remaining on the excited PES (arrows branching in Figure 1b). A comeback to the ground all-*trans* state (*BR*_568_) is possible via movement along the excited PES in the backward direction. Within the framework of this crossing model, equality of quantum yields of the forward and reverse photoreactions is expected: *Y*_1_ ≈ *Y*_2_ [69]. To elucidate the mechanism of S_1_ → S_0_ conversion in the CI region, it is important to measure the quantum yield of the *BR* reverse photoreaction. 

**Figure 7:**
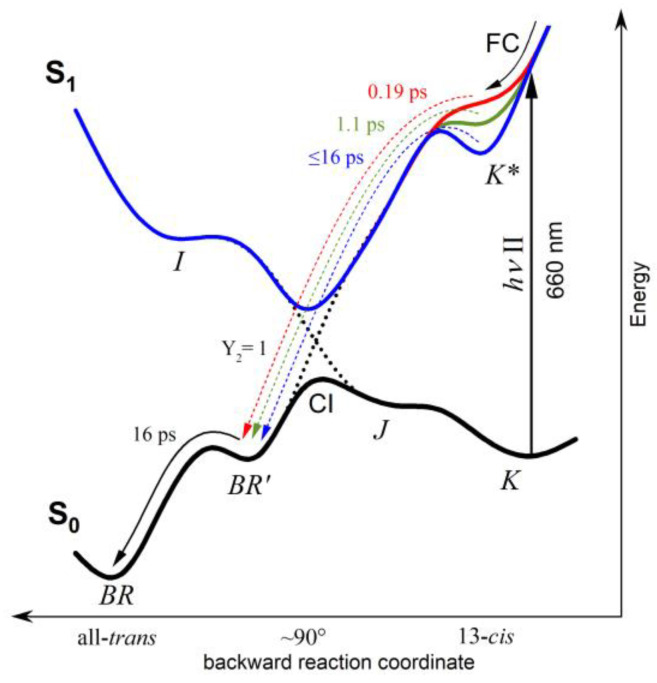
Assumed structure of potential energy surfaces (PESs) of S_0_ and S_1_ electron levels of *BR* participating in the reverse photoreaction. The reaction coordinate is represented by reaction vibrational modes; all-*trans*, 13-*cis,* and intermediate forms of RPSB are marked. Red, green, and blue dashed arrows illustrate concomitant S_1_ → S_0_ transitions of the reverse photoreaction with times of 0.19, 1.1, and 16 ps, respectively. FC—Franck–Condon state; CI—conical intersection.

The zero transient absorption at 45 ps after pulse II (Figure 5) means that the 13-*cis* → all-*trans* transition of the reverse *BR* photoreaction has the same quantum yield *Y*_2_ = 1 as was measured at a nanosecond time scale [8,23,47] and differs substantially from the quantum yield *Y*_1_ = 0.65 of the forward photoreaction. Since the reverse photoreaction was initiated from the product *K*_590_ immediately after its formation, the motion through the CI region during the reverse photoreaction follows via phase space trajectories that differ significantly from the trajectories of the forward reaction (Figure 7). The differences in the reaction pathways of the forward and reverse reactions may be caused by a multifaceted topology of the CI region. Even for the simplest molecule of AlH_2_ consisting of three atoms, the conical intersection represents three points that are not connected to each other [72]. The author concludes that in complex systems, additional intersection regions may appear in unexpected areas of nuclear coordinate space. 

The high quantum yield of the reverse photoreaction means the absence of considerable branching and justifies the usage of the sequential kinetic model (Scheme 1) for the derivation of the difference spectra of intermediates. The reverse photoreaction of *BR* is unexpectedly slow. The characteristic times τ_1_′, τ_2_′, and τ_3_′ are in a good agreement with the data obtained in [45] upon initiation of the reverse photoreaction from the *K*_590_ product at a time delay of 60 ps (0.3, 1.7, and 11 ps). Our data fully confirm the conclusion of [45] that the excited state *K*_590_* is long-lived, finally decaying with a time of > 10 ps. However, our data demonstrate that the primary product of the S_1_ → S_0_ conversion differs significantly from the *BR*_568_ state: the transient spectra in Figure 5 at delays of 1–6 ps contain a significant positive absorption band in the region of 620–700 nm, whereas similar spectra in Figure 6b of ref. [45] are represented in this region by a bleaching band with a minimum at ~620 nm. The differences may be caused, firstly, by the fact that when calculating the number of *BR*_568_ molecules excited by pulse I and then by pulse II, we were able to determine the probabilities of these events (parameters α and β) quite accurately (see Supplementary Materials, Section S1.2); and secondly, by the fact that the number of molecules of product *K*_590_, not excited by pulse II and contributing to the Δ*A*^I+II^(λ, *t*) signal (the second term of Equation (S6)), was taken into account.

One of the reasons for the presence of several excited states with different decay dynamics and product formation efficiencies in rhodopsins during the forward photoreaction may be variations in the structure of their chromophore centers. The main factors are the conformation of RPSB [19,73] and its interaction with charged amino acid residues, primarily with the complex counterion [18,28,39,40,74,75,76,77,78,79,80], in the case of *BR* consisting of the Asp85 (primary counterion/proton acceptor), Asp212, and three water molecules [81]. Disruption of the hydrogen bond system that mediates this interaction can lead to a significant slowdown in the photoreaction and a decrease in its efficiency [28]. For *BR* [18,65,79], proteorhodopsin (GPR) [18], *Krokinobacter eikastus* sodium pump rhodopsin (KR2) [39], and *Chlamydomonas reinhardtii* channelrhodopsin (ChR2) [80], an initial heterogeneity of the chromophore center was revealed, associated with partial protonation of the primary counterion or disruption of the hydrogen bond between the counterion and RPSB. In the case of *BR*, over a wide pH range, the proportion of molecules with protonated Asp85 is extremely small [18], but most likely it determines the presence of a time component τ_2_ = 3.5 ps, characterizing the non-reactive excited state decay. It can be assumed that in the case of the reverse photoreaction of *BR*, the presence of three decay times of the *K*_590_* excited state is determined by the heterogeneity of the chromophore center of this product, which may also be indicated by the wide absorption spectrum of *K*_590_ at time delay of 5 ps (Figure 4b, blue curve). Using FTIR spectroscopy and X-ray structural analysis, it was shown that the formation of the *K*_590_ product weakens or disrupts the hydrogen bond between the RPSB proton and the W402 water molecule, which is part of the complex counterion, and through it with Asp85 and Asp212 [82,83,84,85,86]. Violation of the stable RPSB-counterion system can cause variations in the interaction of the RPSB proton with charged and polar amino acid residues, which will entail a change in the dynamics of the reverse photoreaction. The heterogeneity of the chromophore center of *K*_590_ associated with the protonation of Asp212 was shown using theoretical calculations [45]. Another reason for this heterogeneity may be the difference in the structure of the chromophore center of the products *J*_625_ and *K*_590_, the ratio of which at a time delay of 5 ps was estimated as 30/70. Another suggestion is that the heterogeneity of the excited state may be related to the nature of the population of different vibrational modes during excitation in different molecular ensembles. Photoexcitation of the structurally unrelaxed, twisted 13-*cis* chromophore most likely results in excitation of both reactive and non-reactive vibrational modes. Since the photoreaction of rhodopsins occurs in a coherent regime, the population of some non-reactive vibrational modes may contribute to the loss of coherence of reactive vibrational modes and thereby slow down the reverse photoreaction.

Photoisomerization of RPSB from *cis* to *trans* form in the same environment usually occurs faster than the *trans* → *cis* phototransition [3,19,73]. The τ_1_′ time obtained in the present work is in good agreement with this pattern. It can be assumed that in about 20% of *K*_590_ molecules, the interaction of RPSB with the complex counterion is not significantly disturbed, while in 80% of the molecules, significant changes have occurred in this interaction. Based on the obtained data, the following scheme of PESs of the reverse photoreaction of *BR* can be presented (Figure 7). The photoreaction is initiated in three fractions of molecules. The initial dynamics of the wave packet, as a result of which the *K*_590_* excited state is formed, most likely does not differ in these fractions. The time of the subsequent S_1_ → S_0_ transition strongly depends on the interaction of RPSB with the protein environment, which can affect both the shape of the PES of the S_1_ electron level and the structure of CI. Because the gas phase *cis* → *trans* photoisomerization of RPSB occurs in 0.4 ps [87], it can be concluded that the protein environment can not only accelerate, as evidenced by the time component of 0.19 ps, but also slow down this process. In 80% of *K*_590_ molecules, the reverse photoreaction is significantly slower (1–16 ps) compared to the gas phase. The vibrational relaxation processes during the formation of the products of the reverse photoreaction take much longer than in the forward photoreaction (16 ps compared to 3.5 ps).

## 4. Materials and Methods

Reagents were purchased from Sigma, Panreac (Barcelona, Spain) and Anatrace (Maumee, OH, USA).

### 4.1. Preparation of BR Samples

Purple membranes were obtained from *H. salinarum* ET1001 cells and kindly provided by the Central Research Technological Institute “Tekhnomash”. A suspension of purple membranes in Na-phosphate buffer (25 mM, pH 7.0) was sonicated at 90 W for three minutes to reduce light scattering. In samples prepared for femtosecond spectroscopy experiments, the concentration of *BR* was 1.8 mg/mL, the purity factor (*A*_280_/*A*_568_) was 1.78, and the absorption at the maximum of the α-band was 4.4 OD units/1 cm optical length. Light adaptation was achieved by illuminating with a halogen lamp (KGM24-250, 24 V, 250 W, Ufa Electric Lamp Plant, Ufa, Republic of Bashkortostan, Russia) for three minutes.

### 4.2. Stationary Spectroscopy

Stationary absorption spectra of *BR* were recorded using a UV 1700 spectrophotometer (Shimadzu Corporation, Kyoto, Japan) and quartz cuvettes with an optical length of 1 cm. For this purpose, *BR* samples prepared for femtosecond experiments were diluted 10 times. This consisted of a sonicated suspension of purple membranes in a Na-phosphate buffer (25 mM, pH 7.0) containing *BR* at a concentration of 0.18 mg/mL and an absorption at the maximum of the α-band of 0.44 OD units/1 cm optical length.

### 4.3. Femtosecond Transient Absorption Spectroscopy

The photoinduced absorption signals of *BR* (∆*A*(λ, *t*)) were recorded on a femtosecond setup using the pump-probe [88] and pump-pump-probe [44] methods. The forward photoreaction of *BR* was initiated by pulse I with a wavelength of 540 nm, a duration of 20 fs, a diameter in the sample of 300 μm, and an energy of 200 nJ (Figure 4b, black curve). The reverse photoreaction of *BR* was initiated by pulse II with a wavelength of 660 nm, a duration of 25 fs, a diameter in the sample of 180 μm, and an energy of 200 nJ (Figure 4b, grey curve). Excitation of *BR* by pulse II occurred at a time delay of *t*_1_ = 5 ps after pulse I; excitation by pulse II without preliminary excitation by pulse I was also used for control. Pulses I and II were linearly polarized parallel to each other, and their density was no more than 3·10^15^ photon/cm^2^, which corresponded to the linear regime with absorption of less than one photon by *BR*_568_ and *K*_590_ molecules. For probing, we used a supercontinuum pulse with an energy of 10 nJ, a diameter in the sample of 100 μm, a time delay relative to pulse I of up to 12 ps, and linear polarization at a “magic” angle of 54.7° relative to pulses I and II. The experiments were carried out at a temperature of 21 °C in a flow optical cell with an optical path length of 0.5 mm. The circulation rate in the flow cell was 9 mL/min, which excluded multiple excitations of the same sample volume at the used excitation frequency of 60 Hz. The spectra were corrected by group delay dispersion as described previously [89]. Accounting for the coherent artifact and deconvolution of the hardware function in the region of *t* = 0 were carried out in accordance with the method described in [90,91]. The difference spectra of *BR* absorption Δ*A*^I^(λ, *t*) and Δ*A*^I+II^(λ, *t*), recorded after the action of pulses I and I + II, respectively, were obtained by accumulating 50 signals. Additionally, the spectra Δ*A*^I^(λ, *t*), Δ*A*^I+II^(λ, *t*), and Δ*A*^II^(λ, *t*) were recorded at a time delay of *t*_2_ = 50 ps with an accumulation of 5000 signals. Based on the obtained data, the spectral-temporal matrix Δ*A*_3_(λ, *t*—*t*_1_) was calculated, characterizing the dynamics of the reverse photoreaction *K*_590_ → *BR*_568_, where *t*—*t*_1_ was the time delay relative to pulse II (see Supplementary Materials, Equations (S1)–(S7)). The Matlab application package https://www.mathworks.com/products/matlab.html (accessed on 01 August 2024) was used to process the data. 

### 4.4. Mathematical Analysis of Absorption Dynamics

The absorption dynamics of the forward and reverse *BR* photoreactions was analyzed by decomposing the spectral-temporal matrices Δ*A*^I^(λ, *t*) and Δ*A*_3_(λ, *t*—*t*_1_) into a linear combination of *n* discrete exponential functions:

(1) $$\Delta A\left(\lambda ,t\right)=\sum_{k=1}^{n}D_{k}(\lambda )\cdot e^{-t/\tau_{k}}+A_{n+1}(\lambda ),$$

under the assumption that the characteristic times {τ_k_} can be considered as “global” parameters, and the wavelength-dependent pre-exponential amplitudes *D*_k_(λ) represent the spectra associated with exponential decay (decay-associated difference spectra, DADS) [92]. The times {τ_k_} were found by a nonlinear minimization procedure of the normalized sum of squared residuals, the amplitudes *D*_k_(λ), and the final spectrum *A*_n+1_(λ), were calculated using the standard linear regression method [93]. 

If the spectral dynamics can be approximated by a system of *n* linear differential equations describing transitions between *n* + 1 electronic states, then the solution of such a kinetic model will be a linear combination of *n* exponential components, with the decay times *τ_i_* being defined as the roots of the characteristic polynomial of the system of linear differential equations. In most cases, quantitative evaluation of the number *n*, the amplitudes *D_k_*(*λ*), and the decay times *τ_k_* of the decomposition (1) are mathematically reliable [94]. However, it is impossible to determine, in a common case, the kinetic model and the respective matrix of transition rates from the experimental data without additional assumptions, since any rotation of the state vector by an orthogonal transformation does not change the characteristic roots [95]. In other words, a single set of DADS in Equation (1) corresponds to an infinite number of different kinetic models, the choice between which can be made only on the basis of additional information [96]. 

In the simplest case considered here, the DADS components were enumerated in accordance with increasing decay times and considered formally as *n* sequential kinetic transitions (Scheme 1).

After determining the characteristic times {τ_k_}, the difference spectra *A*_k_(λ) of the intermediate products (which are also called evolution-associated difference spectra, or EADS, in the sequential kinetic model [92]) were calculated by the inverse of the transition rate matrix. If the decay times differ significantly, *τ*_1_ << *τ*_2_ <<…<< *τ*_n_, the EADS of the intermediates are expressed through the DADS components in simple terms:

(2) $$A_{k}\left(\lambda \right)=\sum_{i=k}^{n}D_{i}(\lambda )+A_{n+1}(\lambda ).$$

Although structural and kinetic heterogeneities, including the trajectory branching in the CI region [97], are inherent for the photochemistry of *BR*, its kinetic transitions can be analyzed in the first approximation within the framework of a linear sequential model [19]. 

## 5. Conclusions

In this work, the dynamics of the reverse photoreaction of *BR* initiated from the product *K*_590_ at an early stage of its formation was characterized. It was shown that the reverse photoisomerization of the RPSB chromophore occurs through an excited state with times of 0.19, 1.1, and 16 ps with a quantum yield of ~1. *BR*_568,_ as the final product of the reverse photoreaction, is formed from intermediate states undergoing vibrational relaxation. The presence of three times of the excited state decay can be determined by the presence of different potential barriers on the potential surface of the excited state, which control the rate of intramolecular S_1_ → S_0_ conversion of the *K*_590_* excited state to the primary vibrationally excited product *BR*′. Comparison with the dynamics of the forward photoreaction showed that the reverse photoreaction proceeds, as a whole, more slowly due to a significant increase in the proportion of picosecond components of the excited state decay. At the same time, the reverse dynamics are faster and more efficient in the region of CI: the lifetime of the fastest component of S_1_ → S_0_ conversion is 0.19 vs. 0.52 ps, and the quantum yield is 1 vs. 0.64. Thus, the obtained results may indicate that the interaction of the chromophore with the protein environment in *BR* during the forward photoreaction at the stage of the *K*_590_ product becomes heterogeneous. 

## Figures and Tables

**Table 1:** Comparison of parameters of the DADS and EADS obtained for the forward and reverse *BR* photoreactions. Lifetimes (τ_n_) and corresponding excited state absorption intensities (ESA1) obtained by decomposing the EADS into Gaussian components.

*BR*_568_ → *K*_590_	τ_0_, ps	τ_1_, ps	τ_2_, ps	
	0.08	0.52	3.5	
	ESA1*_I_*(474 nm), %			
		91	9	
*K*_590_ → *BR*_568_		τ_1_′, ps	τ_2_′, ps	τ_3_′, ps
		0.19	1.1	16
	ESA1_K_*(460 nm), %			
		20	60	20

## Data Availability

Datasets of transient absorption matrices are available on request from the authors.

## Supplementary Materials

The following supporting information can be downloaded at: https://www.mdpi.com/article/10.3390/molecules29204847/s1, Figure S1: spectral intermediates of forward *BR* photoreaction, Figure S2: spectral intermediates of reverse *BR* photoreaction, Table S1: statistical characterization of forward and reverse *BR* photodynamic ensembles.
