# Supplementary material for: Reversible Photochromic Reactions of Bacteriorhodopsin from Halobacterium salinarum at Femto- and Picosecond Times
Source: Molecules. 2024 Oct 13;29(20):4847. doi: 10.3390/molecules29204847 (PMC11510181; doi:10.3390/molecules29204847)
Supplement: Supplementary file 1 [file molecules-29-04847-s001.zip › molecules-3206729-supplementary.pdf]

SUPPLEMENTARY MATERIALS

Reversible photochromic reactions of bacteriorhodopsin from *Halobacterium salinarum* at femto- and picosecond times

Olga Smitienko, Tatyana Feldman, Ivan Shelaev, Fedor Gostev, Arseniy Aybush , Dmitry Cherepanov , Victor Nadtochenko, Mikhail Ostrovsky

S1 Analysis of signal $\Delta A^{I+II}(\lambda, t)$

S1.1 Calculation of the spectral-temporal matrix $\Delta A_3(\lambda, t - t_1)$ characterizing the dynamics of the reverse photoreaction of *BR*

The absorption dynamics of signal $\Delta A^{I}(\lambda, t)$ is caused by changes in the electronic properties of $N_0 = \alpha \cdot N$ molecules of BR_{568} excited by pulse I, where N is the total number of BR_{568} molecules in the volume of the flow system through which the probe pulse passes, and α is the probability of excitation of an individual molecule. Since, during the forward photoreaction, some of the excited molecules passing through the CI region return to the ground state of BR_{568}, the total number of molecules whose state differs from BR_{568} after the action of pulse I will be equal to:

$$N^{I}(t) = N_0 \cdot \eta_1(t) = \alpha \cdot N \cdot \eta_1(t), \qquad \text{(S1)}$$

where $\eta_1(t)$ is a dynamic variable characterizing the probability of formation of all states different from BR_{568} after the action of pulse I. Among these states, one can distinguish the initial excited FC state, the excited state after relaxation of the nuclei I_{460}, as well as the products J_{625} and K_{590}. The function $\eta_1(t)$ changes from 1 at $t = 0$ to $\eta_1(t_2) = \eta_1 = 0.64$ in accordance with the quantum yield of the forward photoreaction ($t_2 = 50$ ps). The absorption changes $\Delta A^{I+II}(\lambda, t)$ after the action of pulse II can be represented as:

$$\Delta A^{I+II}(\lambda, t) = \Delta A_1(\lambda, t) + \Delta A_2(\lambda, t - t_1) + \Delta A_3(\lambda, t - t_1), \qquad \text{(S2)}$$

where $t_1 = 5$ ps and the difference spectra $\Delta A_1(\lambda, t)$, $\Delta A_2(\lambda, t - t_1)$, and $\Delta A_3(\lambda, t - t_1)$ reflect signals from three *BR* ensembles: (1) BR_{568} molecules excited by pulse I but not affected by pulse II; (2) BR_{568} molecules excited only by pulse II; (3) K_{590} molecules (and partially J_{625}) excited by pulse II. The evolution of ensembles (1) and (2) in time is determined by the dynamics of the forward photoreaction with time delays t and $t - t_1$ relative to pulse I, respectively; the evolution of ensemble (3) is determined by the dynamics of the reverse photoreaction $K_{590} \rightarrow BR_{568}$ with a time delay $t - t_1$ relative to pulse II.

The absorption dynamics of ensemble (1) can be expressed through the absorption changes $\Delta A^{I}(\lambda, t)$:

$$\Delta A_1(\lambda, t) = (1 - \gamma) \cdot \Delta A^{I}(\lambda, t), \qquad \text{(S3)}$$

where γ is the probability of excitation of K_{590} by pulse II (Table S1).

The number of molecules in ensemble (2) is:

$$N_2(t) = \beta \cdot (N - N^{I}(t_1)) \cdot \eta_1(t - t_1) = \beta \cdot (1 - \alpha \cdot \eta_1(t_1)) \cdot \eta_1(t - t_1) \cdot N, \qquad \text{(S4)}$$

where β is the probability of BR_{568} excitation by pulse II (Table S1). The absorption dynamics of ensemble (2), taking into account Equations (S1) and (S4), can be expressed through the absorption changes $\Delta A^{I}(\lambda, t - t_1)$:

$$\Delta A_2(\lambda, t - t_1) = \zeta \cdot (1 - \alpha \cdot \eta_1(t_1)) \cdot \Delta A^{I}(\lambda, t - t_1), \qquad \text{(S5)}$$

where $\zeta = \beta/\alpha$ is the ratio of the probabilities of BR_{568} excitation by pulses II and I, respectively.

The spectrum of ensemble (3) is represented by states arising in the process of the reverse photoreaction. These are K_{590} molecules in the excited state (K_{590}^{*}), as well as products of the $S_1 \rightarrow S_0$

conversion, other than BR_{568}. The spectra $\Delta A_3(\lambda, t - t_1)$, characterizing the reverse photoreaction at times $t - t_1$ up to 6 ps relative to pulse II, can be calculated using the following Equation:

$$\Delta A_3(\lambda, t - t_1) = \Delta A^{I+II}(\lambda, t) - (1 - \gamma)\cdot\Delta A^{I}(\lambda, t) - \zeta\cdot(1 - \alpha\cdot\eta_1(t_1))\cdot\Delta A^{I}(\lambda, t - t_1). \quad \text{(S6)}$$

The spectrum $\Delta A_3(\lambda, t_2 - t_1)$ can be calculated using Equation (S7):

$$\Delta A_3(\lambda, t_2 - t_1) = \Delta A^{I+II}(\lambda, t_2) - (1 - \gamma)\cdot\Delta A^{I}(\lambda, t_2) - (1 - \alpha\cdot\eta_1)\cdot\Delta A^{II}(\lambda, t_2), \quad \text{(S7)}$$

where the term $(1 - \alpha\cdot\eta_1)\cdot\Delta A^{II}(\lambda, t_2)$ describes the absorption of the ensemble (2).

S1.2 Determination of probabilities α, β, and γ.

To characterize the states arising during the reverse photoreaction, it was necessary to determine the probabilities of excitation of BR_{568} by pulses I and II, as well as the probability of excitation of K_{590} by pulse II (parameters α, β, and γ, respectively) (Table S1). The parameter α was determined by decomposing the EADS with characteristic time τ_1 = 0.52 ps (the I_{460} difference spectrum) (Figure 3b, green curve) into Gaussian components (Figure S1a). This EADS was modeled by the sum of five Gaussian functions with maxima at 400, 474, 526, 652, and 747 nm, and the GSB_{BR} band represented by the absorption spectrum of BR_{568} with the opposite sign. The intensity of this band was estimated by mutual decomposing the difference spectrum $\Delta A^{I}(\lambda, 50\text{ ps})$ (Figure S1c) taking into account that at a delay of 50 ps, the intensity of the GSB_{BR} band was 0.64 of the original one, in accordance with the quantum yield of the forward photoreaction. The final spectrum $\Delta A^{I}(\lambda, 50\text{ ps})$, which represents the difference one of the K_{590} product, was approximated by a Gaussian function with a maximum at 582 nm, a broad scattering band in the short-wavelength region, and the GSB_{BR} band (Figure S1c). The value of α = 0.143 was determined as the ratio of the amplitude of the GSB_{BR} band in the I_{460} difference spectrum (Figure S1a) to the amplitude of BR_{568} absorption in the measuring cell (Table S1).

Table S1. Statistical characterization of forward and reverse *BR* photodynamic ensembles. Values of parameters α, β, γ, and ζ.

	**Pulse I** (BR_{568}) α	**Pulse II** (BR_{568}) β	**Pulse II** (K_{590}) γ	$\zeta = \beta/\alpha$
Excitation probability	0.143	0.028	0.185	0.171

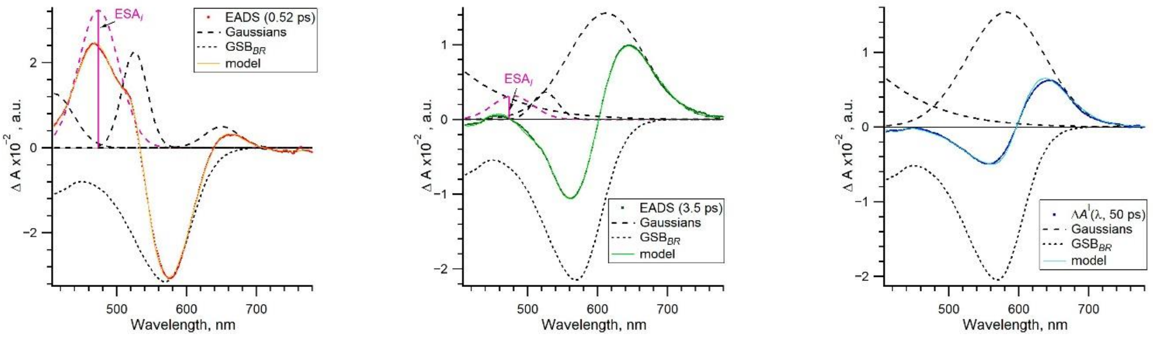

(**a**) (**b**) (**c**)

Figure S1. Spectral intermediates of forward *BR* photoreaction. Decomposition of EADS with characteristic times of 0.52 (**a**) and 3.5 (**b**) ps, and the final spectrum $\Delta A^{I}(\lambda, 50\text{ ps})$ (**c**) into Gaussian components.

The probability β of BR_{568} excitation by pulse II was determined from the ratio of the absorption spectra produced by pulses I and II, respectively: $\zeta = \beta/\alpha = \Delta A^{II}(\lambda, 50\text{ ps})/\Delta A^{I}(\lambda, 50\text{ ps})$. As follows from Figure 4a, the ratio ζ = 0.171; consequently, the probability of BR_{568} excitation by pulse II was β = 0.028.

The ratio of the probabilities of excitation of BR_{568} by pulse I and K_{590} by pulse II can be expressed as:

$$\frac{\alpha}{\gamma} = \frac{\int \varepsilon_{BR}(\lambda)\cdot E_I(\lambda)d\lambda}{\int \varepsilon_K(\lambda)\cdot E_{II}(\lambda)d\lambda} \cdot \frac{E_I \cdot E_{phII} \cdot D_{II}^2}{E_{II} \cdot E_{phI} \cdot D_I^2}, \quad (S8)$$

where $\varepsilon_{BR}(\lambda)$ and $\varepsilon_K(\lambda)$ – the extinction coefficients of BR_{568} and K_{590}, $E_I(\lambda)$ and $E_{II}(\lambda)$ – the spectra of pulses I and II, E_I and E_{II} – the energies of pulses I and II, E_{phI} and E_{phII} – the average energies of photons in pulses I and II, D_I and D_{II} – the diameters of pulses I and II in the sample. The ratio of the overlap integrals of the BR_{568} spectrum with the spectrum of pulse I and the K_{590} spectrum with the spectrum of pulse II was determined from the spectra shown in Figure 4b; this value was 2.62. Based on Equation (S8), the parameter γ was calculated to be 0.185 (Table S1).

To determine the contribution of the non-reactive pathway to the overall dynamics of the I_{460} decay, EADS with characteristic times τ_1 = 0.52 ps and τ_2 = 3.5 ps were decomposed into Gaussian components. Decomposition of EADS (0.52 ps) was performed earlier (Figure S1a). The EADS (3.5 ps) was modeled by the sum of four Gaussian functions with maxima at < 400 (a broad scattering band), 480, 525, and 613 nm, and the GSB_{BR} band (Figure S1b). The contribution of the non-reactive pathway was determined as the ratio of the amplitude of the $ESA1_I$ band at wavelength of 474 nm presented in Figure S1b (pink stick) to the analogous amplitude presented in Figure S1a (pink stick). It amounted to 9% and the contribution of the reactive excited state was 91% accordingly (Table 1).

S2 Decomposition of EADS characterizing the reverse *BR* photoreaction into Gaussian components

EADS with characteristic times τ'_1 = 0.19 ps, τ'_2 = 1.1 ps, and τ'_3 = 16 ps (Figure 6b), obtained by analyzing the $\Delta A_3(\lambda, t - t_1)$ spectra, were decomposed into Gaussian components (Figure S2). As a result, the K_{590}^*, BR'_1, and BR'_2 spectra were obtained (Figure 6c). When decomposing EADS (0.19 ps) (Figure S2a), the linear absorption spectrum of BR_{568}, multiplied by the factor $\gamma \cdot \alpha \cdot \eta_1$ (Figure 6c, black curve), was used.

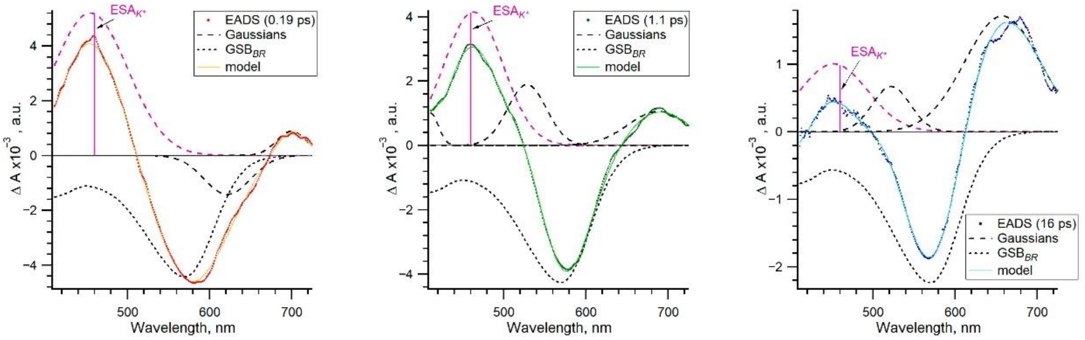

(**a**) (**b**) (**c**)

Figure S2. Spectral intermediates of reverse *BR* photoreaction. Decomposition of EADS with characteristic times of 0.19 (**a**), 1.1 (**b**), and 16 (**c**) ps into Gaussian components.

The ratio of the different paths of the excited state decay was determined from the change in the $ESA1_{K^*}$ signal at a wavelength of 460 nm (Figure S2, pink sticks), considering that the $ESA1_{K^*}$ signal presented in Figure S2a is 100%. These values are presented in Table 1.
